# Automatic Parking Trajectory Planning Based on Warm Start Nonlinear Dynamic Optimization

**DOI:** 10.3390/s25010112

**Published:** 2024-12-27

**Authors:** Hongbin Ren, Yaqi Niu, Yunong Li, Lin Yang, Hongliang Gao

**Affiliations:** 1 State Key Laboratory of Mechanical Transmission for Advanced Equipments, Chongqing University, Chongqing 400044, China; renhongbin2106@126.com; 2 School of Mechanical Engineering, Beijing Institute of Technology, Beijing 100081, China; 3 Xi’an Aerospace Chemical Propulsion Co., Ltd., Xi’an 710089, China

**Keywords:** Hybrid A*, nonlinear optimization, warm start, automatic parking, trajectory planning

## Abstract

In this paper, we propose an optimal parking path planning method based on numerical solving, which leverages the concept of the distance between convex sets. The obstacle avoidance constraints were transformed into continuous, smooth nonlinear constraints using the Lagrange dual function. This approach enables the determination of a globally optimal parking path while satisfying vehicular kinematic constraints. To address the inefficiency typically associated with numerical solving, a warm start strategy was employed for the optimization variables: first, the Hybrid A* algorithm was utilized to generate the initial path values; next, a velocity planning problem was formulated to obtain initial velocity values; and finally, converted convex optimization problems were used to compute the initial dual variables. The optimality of the proposed method was validated through a real car test with ACADO as a solver in three typical parking scenarios. The results demonstrate that the proposed method achieved smoother parking paths in real time.

## 1. Introduction

For automatic parking, whether the vehicle is located on the side of a street or in a designated parking lot, it needs to complete parallel, perpendicular, or angled parking maneuvers within a limited space. Unlike path planning on structured roads, automatic parking lacks a predefined reference path. As a result, the vehicle needs to independently plan a smooth, easily trackable trajectory with minimal error tolerance in relatively confined spaces, enabling the target vehicle to reach a precise location within a certain time frame. In the field of trajectory planning algorithms for automatic parking, many automotive companies and researchers worldwide have already made significant progress. Early traditional planning algorithms are mostly based on geometric methods, such as Dubins curves and Reed–Shepp curves [1,2], which splice straight lines and circular arcs to plan for the shortest path. However, the paths generated by these methods exhibit discontinuities in curvature. Subsequent scholars have tried optimizations with Bezier curves [3], inverse tangent curve fitting [4], quintic polynomials [5], and spline curve [6], refining the parking process and smoothing out the segments and transitions. Although these methods are computationally efficient for specific scenarios, their limited robustness necessitates distinct trajectory modeling for different parking environments, making them challenging to apply in more complex scenarios.

Current trajectory planning algorithms can be divided into three main categories: search-based, sampling-based, and numerical-optimization-based. Classic search-based algorithms like Dijkstra’s algorithm and A* [7] are widely used for shortest path calculations. Dijkstra’s algorithm employs a breadth-first search algorithm, while A* introduces heuristic costs on top of Dijkstra, speeding up the search as a depth-first search algorithm. However, these two methods are computationally expensive and inefficient in complex environments, whereas sampling-based algorithms can better avoid such drawbacks, with classic methods like the probabilistic roadmap method (PRM) [8] and rapid exploration random tree (RRT) [9]. Many scholars have optimized and improved upon these methods, such as ARA* [10] and Bidirectional RRT* [11]. For parking conditions, the Hybrid A* algorithm [12] based on A* has greater advantages because it considers both path optimality and vehicle kinematics, accounting for critical reverse path planning.

Numerical optimization methods in path planning can be divided into two types: one focusing on optimizing and smoothing an initially obtained rough path without considering the vehicle model, and the other that incorporates the vehicle model, transforming the trajectory optimization process into an optimal control problem where the vehicle navigates from the start to the end point without colliding with obstacles. The latter is currently the primary research focus and presents significant challenges, as the constraints related to the vehicle model and obstacle avoidance are typically non-convex and nonlinear, posing considerable difficulties for numerical solvers. Zhang et al. [13] proposed a novel approach to transform non-differentiable obstacle avoidance constraints into smooth nonlinear constraints based on the concept of distance between convex sets. He et al. [14] introduced temporal warm start and dual-variable warm start on this basis, reconstructing the optimization problem and making safety distance an optimization variable as well, reducing unnecessary parameter tuning. Zhang et al. [15] divided the entire time interval of the parking trajectory into K segments, each using Radau pseudo-spectral method to construct a nonlinear programming problem. Additionally, with the development of artificial intelligence technology, deep reinforcement learning [16,17,18,19] has also been applied to path planning. Such methods learn from given parking routes as samples and directly output waypoints. However, they are heavily reliant on perception and exhibit limited adaptability.

**Figure 1:**
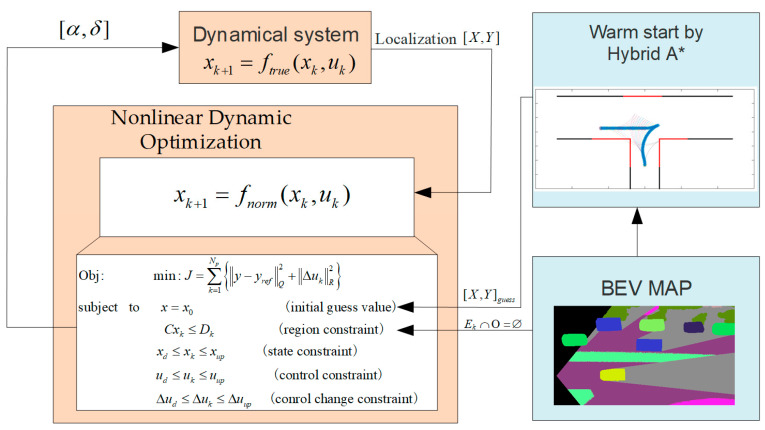
The diagram of warm start nonlinear dynamic optimization for automatic parking.

This paper proposes a method for obtaining the optimal trajectory by solving the optimal control problem. To reduce the impact of low efficiency in numerical solving and to speed up the computation process, there are considerations including path, velocity, and dual variables warm starting for the optimization problem. The diagram of warm start nonlinear dynamic optimization for automatic parking trajectory planning is shown in Figure 1.

The contributions of this paper are summarized as follows: (1) The warm start for the optimization variables is proposed to improve the efficiency of solving the optimization problem. (2) The trajectory planning problem has been transformed into an optimal control problem capable of producing a globally optimal solution.

The paper is organized as follows: The optimization problem is described in Section 2. The warm start approach is proposed in Section 3. In Section 4, “Experimental Verification”, a real-time simulation platform is designed, and three typical parking scenarios are validated. Results analysis is also presented. Finally, conclusions are provided in Section 5. 

## 2. Description and Construction of the Optimization Problem

### 2.1. Vehicle Motion Constraints

**Figure 2:**
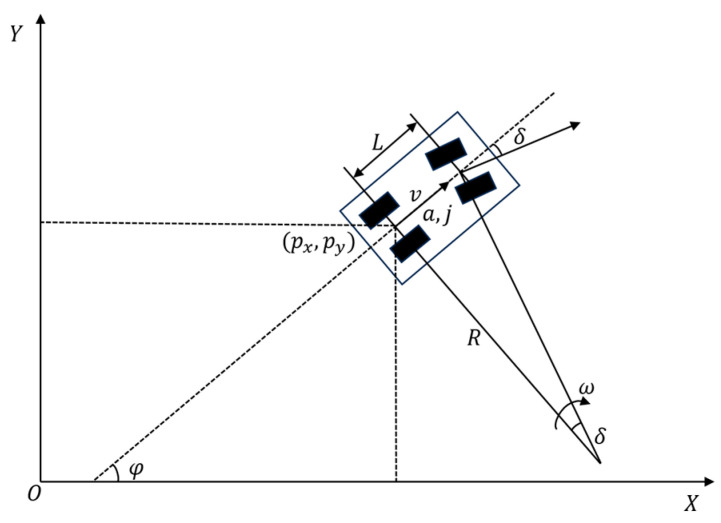
Vehicle kinematic model.

Based on the ideal Ackermann steering model and ignoring tire slip angle, the four-wheel vehicle model can be simplified to a two-wheeled bicycle model as shown in Figure 2, where L represents the wheelbase of the vehicle; v,a,j represent the velocity, acceleration, and jerk of the vehicle’s rear axle center; $\left(p_{x},p_{y}\right)$ represents the position of the rear axle center; δ is the front wheel steering angle; φ is the vehicle’s heading angle; and ω is the angular velocity of the vehicle’s front wheel steering. The state variable is $x={\left[p_{x},p_{y},\varphi ,v,a,\delta \right]}^{T}$, the control variable is $u={\left[j,\omega \right]}^{T}$, and the state equation is

(1) $$\dot{x}={\left[\begin{array}{cccc} v\cdot \cos\varphi & v\cdot \sin\varphi & v\cdot \tan\delta /L & \begin{array}{ccc} a & j & \omega \end{array} \end{array}\right]}^{T}$$

By setting a fixed computation step size and discretizing the state variables using the second-order Runge–Kutta method, the vehicle kinematic equation constraints are derived.

(2) $$x_{k+1}=\left[\begin{array}{c} p_{x_{k}}+dt\cdot \left(v_{k}+0.5dt\cdot a_{k}\right)\cdot \cos\left(\varphi_{k}+0.5dt\cdot v_{k}\cdot \tan\delta_{k}/L\right)\\ p_{y_{k}}+dt\cdot \left(v_{k}+0.5dt\cdot a_{k}\right)\cdot \sin\left(\varphi_{k}+0.5dt\cdot v_{k}\cdot \tan\delta_{k}/L\right)\\ \varphi_{k}+dt\cdot \left(v_{k}+0.5dt\cdot a_{k}\right)\cdot \tan\delta_{k}/L\\ v_{k}+dt\cdot \left(a_{k}+0.5dt\cdot j_{k}\right)\\ a_{k}+dt\cdot j_{k}\\ \delta_{k}+dt\cdot \omega_{k} \end{array}\right]$$

When the fixed time step dt is changed to a variable time step τ(k), the motion equation becomes closer to the actual manual control process. At the same time, it should satisfy the physical constraints of real vehicle parameters such as velocity, acceleration, jerk, yaw rate, wheel steering angle, and its angular velocity. The constraint equation is $\underline{h}\leq h(x_{k},u_{k})\leq \bar{h}$, where $h={\left[v(t),a(t),j(t),\dot{\varphi }(t),\delta (t),\omega (t)\right]}^{T}$.

### 2.2. Obstacle Avoidance Constraints

Avoiding collisions with obstacles is a major focus and difficulty in planning problems. Currently, most research on trajectory planning for obstacle avoidance treats vehicles as a point and incorporates avoidance constraints after processing obstacle inflation. However, this method may lose some optimality. Refs. [20,21] introduced a concept of potential fields to achieve obstacle avoidance by minimizing a potential energy function. The vehicle is modeled as a rectangle to address these shortcomings. For parking problems, obstacles generally manifest as parking spaces occupied by other vehicles, or more strictly, all parking spaces except the target space can be considered. Since in actual parking scenarios, when a single car is given priority to park, nearby vehicles and pedestrians tend to “actively” give way, and trajectory planning near parking spaces can disregard dynamic obstacles to some extent. We consider the obstacle in the environment as a polyhedra constructed by the intersection of a finite number of half-spaces and hyperplanes. Non-convex obstacles can be decomposed into combinations of multiple convex hulls or have their non-convex parts ignored after obstacle inflation. Assuming there are M obstacles in the environment, since the space occupied by an obstacle is convex, each space occupied by an obstacle can be represented as $O^{\left(m\right)}=\{x\in R^{2}|A^{\left(m\right)}x\underline{≺}b^{\left(m\right)}\}(m=1,2,3\ldots M)$. At state $x_{k}$, the space occupied by the vehicle is $E_{k}$, which can be obtained from the vehicle’s initial pose through rotation and translation, i.e., $E_{k}=R(x_{k})E_{0}+t(x_{k})$, where $R(x_{k})$ is the rotation matrix, $t(x_{k})$ is the translation vector, and $E_{0}=\{x\in R^{2}|Gx\underline{≺}g\}$ is the convex space occupied by the vehicle at its initial position. To ensure that the vehicle does not collide with any obstacle at state $x_{k}$, $E_{k}\cap O^{\left(m\right)}=\emptyset (\forall m=1,2,3\ldots M)$ must be ensured.

The above description can also be rephrased as follows: For any point e inside $E_{k}$ and any point $o^{\left(m\right)}$ inside each obstacle, the minimum distance between them should be greater than the safety distance $d_{min}$. The point e can be obtained from the corresponding point in the initial state $e^{\prime }$ through rotation and translation. To avoid collisions with obstacles, there should be the following constraint for any vehicle states and any obstacle.

(3) $$\begin{array}{c} \underset{e^{\prime },o^{\left(m\right)}}{min}\{\left.\left\|R\left(x_{k}\right)e^{\prime }+t(x_{k})-o^{\left(m\right)}\right\|\;\right|A^{\left(m\right)}o^{\left(m\right)}\underline{≺}b^{\left(m\right)},Ge^{\prime }\underline{≺}g\}\geq d_{\min}\\ \forall m=1,2,3\ldots M,\forall k=1,2,3\ldots N \end{array}$$

The left-hand side of Equation (3) constitutes an optimization problem:

(4) $$\begin{array}{l} \underset{e^{\prime },o^{\left(m\right)},\omega }{min}\left\|\omega \right\|\\ \;\begin{array}{c} \begin{array}{ll} s.t. & A^{\left(m\right)}o^{\left(m\right)}\underline{≺}b^{\left(m\right)}\\ & Ge^{\prime }\underline{≺}g\\ & \omega =R\left(x_{k}\right)e^{\prime }+t(x_{k})-o^{\left(m\right)} \end{array} \end{array} \end{array}$$

Consider the Lagrange dual function of the problem in Equation (4):

(5) $$\begin{array}{ll} g\left(\lambda ,\mu ,z\right)= & \underset{e^{\prime },o^{\left(m\right)},\omega }{inf}\left(\begin{array}{l} \left\|\omega \right\|+{\left(A^{\left(m\right)}o^{\left(m\right)}-b^{\left(m\right)}\right)}^{T}\lambda +{\left(Ge^{\prime }-g\right)}^{T}\mu \\ +z^{T}\left(R\left(x_{k}\right)e^{\prime }+t(x_{k})-o^{\left(m\right)}-\omega \right) \end{array}\right)\\ & =\left\{\begin{array}{ll} -g^{T}\mu +zt\left(x_{k}\right)-b^{T}\lambda & \;\;\;\lambda^{T}A=z^{T}\\ \;+\underset{\omega }{\sup}\left(z^{T}\omega -\|\omega \|\right) & \;\;\;\mu^{T}G+z^{T}R\left(x_{k}\right)=0\\ \;-\infty & \;\;\;\;\;else\; \end{array}\right. \end{array}$$

λ and μ in Equation (5) are both Lagrange multipliers corresponding to the inequality constraints, and thus they must satisfy λ≥0,μ≥0. For the term involving $\;sup\left(z^{T}\omega -\left\|\omega \right\|\right)$ in the equation, it can be simplified using the dual norm shown in Equation (6).

(6) $$\begin{array}{l} \underset{\omega }{sup}\left(z^{T}\omega -\left\|\omega \right\|\right)=\underset{\omega }{sup}\left\|\omega \right\|\cdot \underset{\omega }{\;sup}\left({\left(\frac{\omega }{\left\|\omega \right\|}\right)}^{T}z-1\right)\\ \overset{{\left\|v\right\|}_{*}=\underset{\left\|u\right\|\leq 1}{sup}u^{T}v}{\to }=\underset{\omega }{sup}\omega \cdot \left({\left\|z\right\|}_{*}-1\right)=\left\{\begin{array}{l} 0\;{\left\|z\right\|}_{*}\leq 1\\ \infty \;\;else \end{array}\right. \end{array}$$

In Equation (6), ${\left\|z\right\|}_{*}$ is the dual norm of $\;\left\|z\right\|$. Then, Equation (5) can be rewritten as

(7) $$g\left(\lambda ,\mu ,z\right)=\left\{\begin{array}{cc} -g^{T}\mu +zt(x_{k})-b^{\left(m\right)T}\lambda & if\;\left\{\begin{array}{c} \lambda^{T}A^{\left(m\right)}=z^{T}\\ \mu^{T}G+z^{T}R\left(x_{k}\right)=0\\ {\left\|z\right\|}_{*}\leq 1 \end{array}\right.\\ -\infty & else \end{array}\right.$$

It is easy to know from the definition of the dual norm that the value of ${\left\|A^{(m)T}\lambda \right\|}_{*}$ equals the maximum absolute value of the elements in vector $A^{(m)T}\lambda$. Using the Cauchy–Schwarz inequality, it can be proven that ${\left\|A^{(m)T}\lambda \right\|}_{*}\leq {\left\|A^{(m)T}\lambda \right\|}_{2}$. By replacing ${\left\|A^{(m)T}\lambda \right\|}_{*}$ with ${\left\|A^{(m)T}\lambda \right\|}_{2}$, a new convex constraint can be constructed under the premise of satisfying the constraints, which is easier to solve. At the same time, since any obstacle $O^{\left(m\right)}$ has a non-empty relative interior, strong duality holds, and the original problem and the dual problem have the same optimal objective value:

(8) $$\underset{e^{\prime },o^{\left(m\right)},\omega }{min}\omega =\underset{\lambda ,\mu ,z}{max}g(\lambda ,\mu ,z)$$

The final description of the obstacle avoidance constraint is shown in Equation (9), where $d_{min}$ is the safety margin, here being a real number greater than 0.

(9) $$\left\{\begin{array}{c} -g^{T}\mu +{\left(A^{\left(m\right)}t(x_{k})-b^{\left(m\right)}\right)}^{T}\lambda \geq d_{min}\\ \mu^{T}G+\lambda^{T}A^{\left(m\right)}R\left(x_{k}\right)=0\\ \;{\left\|A^{{\left(m\right)}^{T}}\lambda \right\|}_{2}\leq 1,\lambda \geq 0,\mu \geq 0 \end{array}\right.$$

### 2.3. Optimization Problem Final Description

In this paper, a cost function was chosen to ensure that during the process from the initial state $x_{0}$ to the target state $x_{F}$, the planned trajectory had higher economy and comfort, under the premise of not colliding with obstacles. Additionally, to accelerate the solution process, the equality constraints on the terminal state are relaxed here, requiring only that the terminal state is within a certain error range ε of the target state. Then, the final NLP (nonlinear programming) problem is obtained as follows:

(10) $$\begin{array}{ll} \underset{\tau ,x,u,\lambda ,\mu }{min} & \overset{N}{\underset{k=0}{\sum }}\left(u_{k}^{T}Q_{u}u_{k}+\Delta u_{k}^{T}\left(k\right)Q_{\Delta u}\Delta u_{k}+Q_{\tau }\tau_{k}\right)\\ s.t. & x_{0}=x_{s},\;\left\|x_{N}-x_{F}\right\|\leq \varepsilon ,\;\varepsilon \geq 0\;\;\;\;\;(10.1)\\ & x_{k+1}=x_{k}+\tau_{k}f\left(x_{k}+0.5\tau_{k}f\left(x_{k},u_{k}\right),u_{k}\right)\;\;\;(10.2)\\ & h\left(x_{k},u_{k}\right)\leq 0\;\;\;\;(10.3)\\ & equation\left(9\right)\;\;\;\;\;\;(10.4) \end{array}$$

In Equation (10), the objective function is selected to minimize the cost of control inputs and their increments, as well as parking time, where $Q_{u},Q_{\Delta u},Q_{\tau }$ represent the corresponding weight matrix. To enhance the efficiency of solving, a variable sampling step length is adopted for optimization. Equation (10.1) specifies the initial and terminal pose constraints, including position and orientation information. Equation (10.2) relates to vehicle kinematic constraints, and Equation (10.3) sets the upper and lower bounds for the quantities being optimized.

## 3. Warm Start for the Optimization Problem

A warm start includes path, velocity, and dual variables to provide the optimization solver with an initial solution that is closer to the optimal point, improving convergence speed and stability. Warm starting these variables is especially effective in iterative problems or scenarios with slight changes in constraints or objectives, such as in model predictive control (MPC) or real-time optimization tasks.

### 3.1. Path Warm Start

The path warm start uses an improved Hybrid A* (HA*) algorithm. HA* is an efficient path planning algorithm that considers the actual motion constraints of a vehicle, and compared to the traditional A* algorithm, it has been improved in aspects such as the path expansion and heuristic function. This paper improves upon the traditional HA* algorithm by providing a complete algorithmic treatment and attempts to connect the endpoint using hybrid curvature (HC) paths, which are more continuous in curvature than Reed–Shepp (RS) paths [22]. The details and theoretical background are shown in Section 3.1.2.

#### 3.1.1. Algorithm Workflow

Firstly, a candidate search area based on the vehicle’s collision avoidance radius and obstacle avoidance constraints was determined using a KDTree search model in order to avoid excessive collision calculations between the ego vehicle and obstacles. Next, a grid-shaped heuristic search was conducted from the endpoint according to the A* algorithm, resulting in a heuristic value function map for every non-obstacle point to the endpoint. Then, combining the vehicle’s motion constraints with the heuristic cost map, nodes were expanded, and the current node with the minimum cost was selected from the expanded nodes. The cost includes the vehicle’s maneuvering such as steering, reversing, U-turns, changes in steering angle, and the heuristic cost. Finally, the shortest HC path is attempted to replace the RS path to connect the current node to the target endpoint, and the curve that satisfies the obstacle avoidance requirements becomes the final planned path.

**Figure 3:**
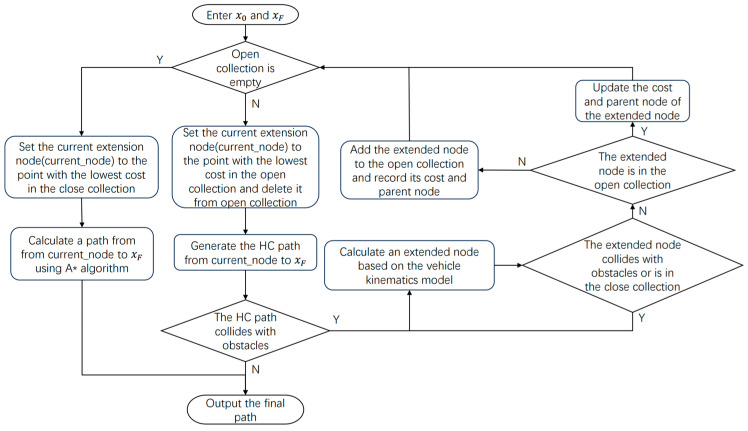
Algorithm workflow of the HA* algorithm.

However, the HA* algorithm is not complete in that the curves obtained from expanded nodes or the HC path may be unsolvable due to environmental constraints, thus losing the advantage of warm starting. Therefore, the improved HA* algorithm in this paper is made more complete and fault-tolerant: if the open set is empty and the final path has not been found, the node with the lowest cost is selected from the closed set to continue the search with the A* algorithm. In this way, even if the latter part of the path does not meet the motion conditions, it can still provide a good initial value compared to a zero-initial value. Overall, the algorithm workflow is shown in Figure 3.

#### 3.1.2. Hybrid Curvature (HC) Paths

**Figure 4:**
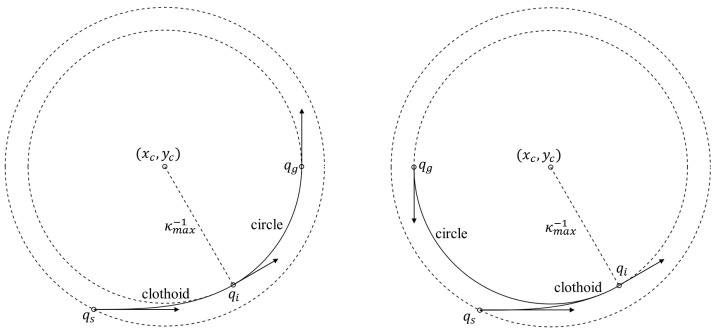
Examples of the HC turn: the regular HC turn is on the left and the irregular HC turn is on the right. $q_{s}$ is the start point of the HC turn, which then leads into the clothoid transition segment; $q_{i}$ is the start point of the circle segment; $q_{g}$ is the end point of the HC turn.

The HC path was first proposed by Holger Banzhaf et al. [20] in 2017, introducing a new type of steering function called hybrid curvature steering based on the continuous curvature (CC) path 21. It ensures that when a vehicle travels in one direction, the generated path has continuous curvature, while allowing for discontinuities in curvature during gear shifts. HC paths consist of HC turns and straight lines. As shown in Figure 4, HC turn starts from a point with zero curvature, enters a clothoid transition segment, and eventually reaches the vehicle’s minimum turning circle. Since the clothoid curve has a linear relationship between curvature and arc length, the resulting HC path has continuous curvature in the same direction segments.

**Figure 5:**
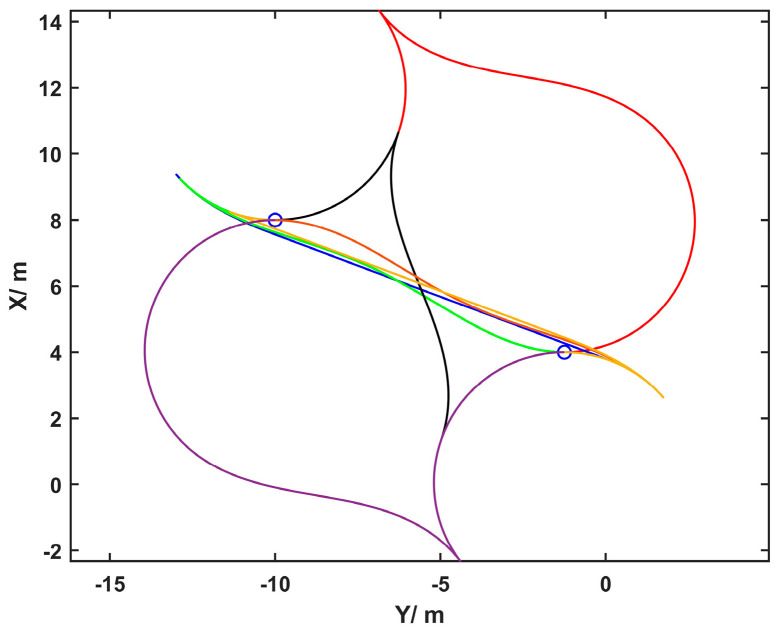
Examples of HC paths connecting two given points.

Like the way that the RS path utilizes circles (C) and straight lines (S) as well as considers nine combinations involving forward and reverse motions, the HC path adds four new combinations based on this, resulting in a total of 13 fundamental HC families shown in Table 1. And Figure 5 provides examples of HC paths connecting two given points.

#### 3.1.3. Collision Detection Method

**Figure 6:**
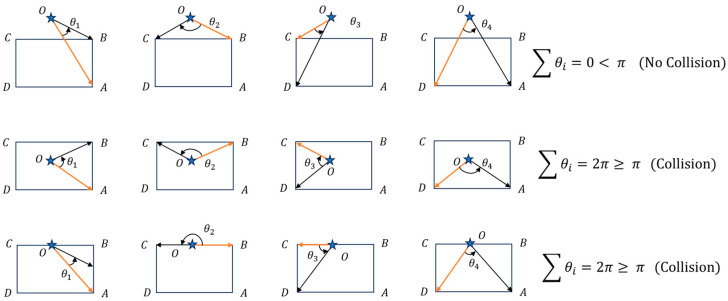
Rectangular collision detection method.

For the collision detection of the paths in the workflow shown in Figure 3, this paper adopted a collision detection method that combines circular collision detection with rectangular collision detection. First, the target vehicle is quickly screened using circular expansion to filter out path segments that will definitively not collide, which speeds up the detection process. Then, for places with interference, rectangular collision detection is performed. The vehicle’s outer contour is treated as a rectangle, and the radius of the circular expansion is the radius of the circumscribed circle of the vehicle. Obstacle points where circular detection indicates interference are referred to as collision points. The rectangular detection uses these collision points as input and determines whether a collision truly exists by calculating angles. The specific method is illustrated in Figure 6, in which the stars represent potential collision points of obstacles. Connecting these potential collision points with the four vertices of the ego vehicle’s rectangle in a counterclockwise sequence, let it be known that when $\vec{OB}$ is on the counterclockwise side of $\vec{OA}$, the angle between two vectors is positive; otherwise, it is negative. If an obstacle point is on the boundary or inside of the vehicle, the sum of the angles is 360°, and if it is outside, the sum of the angles is 0°. The angle between two vectors can be calculated through the dot product. Let the two vectors connecting the detection point with the two endpoints of one side be $\left(x_{1},y_{1}\right)$ and $\left(x_{2},y_{2}\right)$, and the angle between the two vectors θ∈[0,π] is as follows:

(11) $$\theta =\arccos\frac{\left(x_{1}\cdot x_{2}+y_{1}\cdot y_{2}\right)}{\sqrt{\left(x_{1}^{2}+y_{1}^{2}\right)\cdot \left(x_{2}^{2}+y_{2}^{2}\right)}}$$

### 3.2. Velocity Warm Start

**Figure 7:**
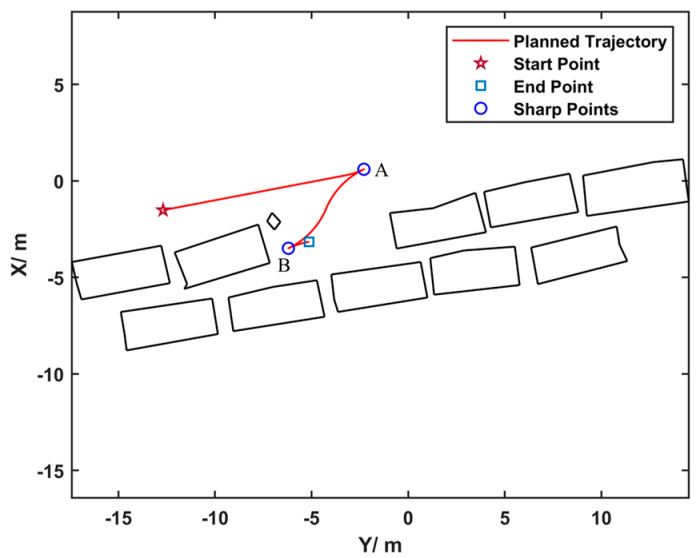
The planning result of HA* for a parking scenario: red is the planned trajectory, black represents obstacles, A and B are “sharp points”.

The velocities calculated from curves that geometrically splice straight lines and arcs often lack smoothness. Considering that mainstream trajectory tracking methods are divided into model predictive-based and geometry-based approaches, the latter is more commonly used in practice. However, in this method, the lookahead distance of either the pure pursuit or the Stanley algorithm is set proportional to vehicle speed, which is determined by the set speed and trajectory curvature. Therefore, a velocity planning problem is performed on the results of the path warm start. The trajectories planned by the HA* algorithm include forward and reverse motions. At the switching points between forward and reverse gears, the vehicle speed should be zero. Hence, the overall trajectory is segmented based on the forward or reverse gear. As shown in Figure 7, labeling points such as A and B are called “sharp points” of the trajectory. Only the switches between forward and reverse gears can result in sharp points, and n sharp points can divide the trajectory into n+1 segments, with velocity planning carried out for each segment separately.

Here, the velocity planning problem aims to obtain a distance–time (s−t) profile with minimum acceleration and jerk along a given path, while constraining velocity, acceleration, and jerk. This leads to a quadratic programming problem in Equation (12).

(12) $$\begin{array}{ll} min & w_{\ddot{s}}\int_{0}^{t_{all}}\ddot{s}{\left(t\right)}^{2}dt+w_{\dddot{s}}\int_{0}^{t_{all}}\dddot{s}{\left(t\right)}^{2}dt\\ s.t. & \dot{s}\left(t\right)\in \;\left[{\dot{s}}_{min},{\dot{s}}_{max}\right]\\ & \ddot{s}\left(t\right)\in \;\left[{\ddot{s}}_{min},{\ddot{s}}_{max}\right]\\ & \dddot{s}\left(t\right)\in \;\left[{\dddot{s}}_{min},{\dddot{s}}_{max}\right] \end{array}$$

First, the segmented trajectory should be discretized. Assuming that each segment’s third derivative of s with respect to t is constant after discretization, there are two methods of discretization: spatial parameter discretization (with a fixed resolution Δs) and temporal parameter discretization (with a fixed resolution Δt). The former potentially resulting in an objective function that is difficult to optimize and constraints that are hard to implement. Therefore, the second method is adopted. The first step is to estimate the total travel time T for the trajectory segment. For a partitioned trajectory segment with a total length of S and zero speeds both at the initial and terminal points, if the vehicle accelerates and decelerates at maximum acceleration and maintains its maximum speed after reaching it (assuming uniform acceleration motion), then for that trajectory segment, the time taken for initial acceleration and final deceleration would be equal to $t_{1}=v_{max}/a_{max}$, and the distance covered during these two phases would be $S_{1}=v_{max}^{2}/(2a_{max})$. The remaining part of the segment travels at a constant speed, taking a time $t_{2}=(S-S_{1})/v_{max}$.

It can be obtained from the above description that the total time taken to traverse this segment of the trajectory is

(13) $$\;T_{init}=2t_{1}+t_{2}=v_{max}/a_{max}+S/v_{max}$$

The calculation process assumes uniform acceleration motion and employs maximum velocity and acceleration, and hence a relaxation factor k(k>1) is introduced to adjust the calculated result, where the estimated total duration is $T=kT_{init}$. With the sampling time Δt, we can obtain discrete points as N=T/Δt. Finally, by adding boundary constraints and continuity constraints, as well as including a reference speed term in the objective function, the original problem can be transformed into the following quadratic programming problem. Solving the QP problem in Equation (14) yields N discrete points corresponding to time t and distance traveled s, with the third derivative of s with respect to t being constant between each pair of discrete points. The vehicle pose corresponding to each sample point can be obtained by interpolating the results from the HA* algorithm, thereby acquiring the final trajectory warm start result.

(14) $$\begin{array}{c} min\;\;w_{\ddot{s}}\overset{N}{\underset{i=1}{\sum }}{{\ddot{s}}_{i}}^{2}+w_{\dddot{s}}\overset{N}{\underset{i=1}{\sum }}{{\dddot{s}}_{i}}^{2}+w_{s_{ref}}\overset{N}{\underset{i=1}{\sum }}{\left({\dot{s}}_{i}-s_{ref}\right)}^{2}\\ \;\;s.t.\;\;\;s_{1}=0,s_{N}=s_{all}\\ \;\;\;\;\;\;\;\;{\dot{s}}_{1}=0,{\dot{s}}_{N}=0\\ {\dot{s}}_{i}\in \left[{\dot{s}}_{min},{\dot{s}}_{max}\right]i=1,\ldots ,N\\ {\ddot{s}}_{i}\in \left[{\ddot{s}}_{min},{\ddot{s}}_{max}\right]i=1,\ldots ,N\\ \;{\dddot{s}}_{i}\in \left[{\dddot{s}}_{min},{\dddot{s}}_{max}\right]i=1,\ldots ,N-1\\ \;{\dddot{s}}_{i}=\frac{\;\;{\ddot{s}}_{i+1}-\;{\ddot{s}}_{i}}{\Delta t}i=1,\ldots ,N-1\\ {\ddot{s}}_{i+1}=\;{\ddot{s}}_{i}+\;{\dddot{s}}_{i}\cdot \Delta ti=1,\ldots ,N-1\\ {\dot{s}}_{i+1}={\dot{s}}_{i}+\;{\ddot{s}}_{i}\cdot \Delta t+\frac{\;{\dddot{s}}_{i}\cdot \Delta t^{2}}{2}i=1,\ldots ,N-1\\ s_{i+1}=s_{i}+{\dot{s}}_{i}\cdot \Delta t+\frac{\;{\ddot{s}}_{i}\cdot \Delta t^{2}}{2}+\frac{\;{\dddot{s}}_{i}\cdot \Delta t^{3}}{6}\;i=1,\ldots ,N-1 \end{array}$$

### 3.3. Dual Variables Warm Start

In the original optimization problem shown as Equation (10), the optimization variables include dual variables λ and μ, which, although they are not the final variables needed for trajectory planning, also require warm start to provide good initial values. It is known that in the original problem, the dual variables for each state $x_{k}$ should satisfy the following constraints:

(15) $$\begin{array}{l} -g^{T}\mu_{k}^{\left(m\right)}+{\left(A^{\left(m\right)}t\left(x_{k}\right)-b^{\left(m\right)}\right)}^{T}\lambda_{k}^{\left(m\right)}\geq d_{min}\\ \begin{array}{c} \;\;\;\;\;\;\;\;\;\mu_{k}^{{\left(m\right)}^{T}}G+\lambda_{k}^{{\left(m\right)}^{T}}A^{\left(m\right)}R\left(x_{k}\right)=0\\ \begin{array}{c} \;\;\;\;\;\;\;\;{\left\|A^{{\left(m\right)}^{T}},\lambda_{k}^{\left(m\right)}\right\|}_{2}\leq 1,\;\lambda \geq 0,\;\mu \geq 0\\ \;\;\;\;\;\;\;\;\;\;\forall m=1,2,3\ldots M,\;\forall k=1,2,3\ldots N \end{array} \end{array} \end{array}$$

The maximum value of $-g^{T}\mu_{k}^{\left(m\right)}+{\left(A^{\left(m\right)}t(x_{k})-b^{\left(m\right)}\right)}^{T}\lambda_{k}^{\left(m\right)}$ in Equation (15) is the optimal solution to the dual problem described in Section 2.2, which also equates to the shortest distance between convex sets. For each point obtained from the path warm start in Section 3.1 corresponding to each obstacle, the initial values for λ and μ must satisfy Equation (15). Therefore, a new optimization problem is formulated to maximize the sum of $-g^{T}\mu_{k}^{\left(m\right)}+{\left(A^{\left(m\right)}t(x_{k})-b^{\left(m\right)}\right)}^{T}\lambda_{k}^{\left(m\right)}$ for each state point corresponding to each obstacle, resulting in the convex quadratic constraint optimization problem shown in Equation (16).

(16) $$\begin{array}{l} \underset{\mu ,\lambda }{min}\sum_{k=0}^{N}\sum_{m=1}^{M}d_{m}\left(k\right)\\ \begin{array}{c} \begin{array}{c} s.t. \end{array}-g^{T}\mu_{k}^{\left(m\right)}+{\left(A^{\left(m\right)}t(x_{k})-b^{\left(m\right)}\right)}^{T}\lambda_{k}^{\left(m\right)}=-d_{m}\left(k\right)\\ \;\mu_{k}^{\left(m\right)T}G+\lambda_{k}^{{\left(m\right)}^{T}}A^{\left(m\right)}R\left(x_{k}\right)=0\\ \;\;\;\;\;\;\;\;\;\;\;\;\;\;\;\;\;\;\;{\left\|A^{{\left(m\right)}^{T}},\lambda_{k}^{\left(m\right)}\right\|}_{2}\leq 1,\;\lambda \geq 0,\;\mu \geq 0,\;d_{m}\left(k\right)\leq 0\\ \forall m=1,2,3\ldots M,\;\forall k=1,2,3\ldots N \end{array} \end{array}$$

By solving the above optimization problem, initial warm start values for the dual variables λ and μ can be obtained. And the value of a dual variable can also indicate whether a constraint is active or inactive at the optimal solution. If the dual variable is zero, the constraint is non-binding, meaning it does not affect the optimal solution; if the dual variable is positive (or negative, depending on convention), the constraint is active and influences the solution.

## 4. Experiment Validation

**Figure 8:**
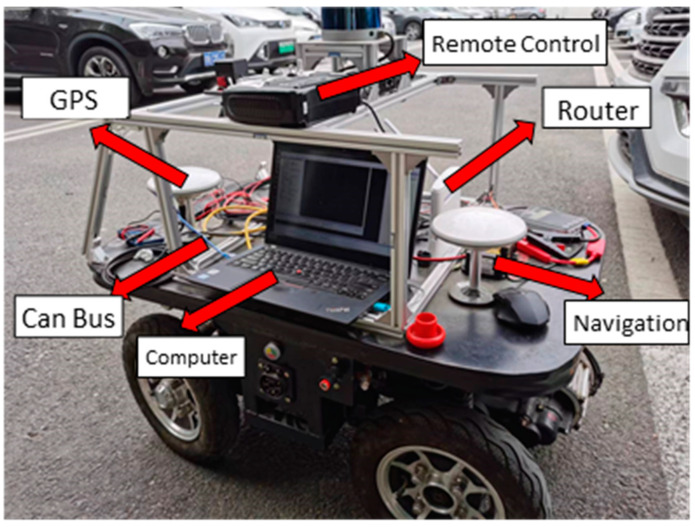
Drive-by-wire platform.

To further validate the effectiveness of the automatic parking algorithm, this paper performed additional tests and verifications of the automatic parking path planning and trajectory tracking algorithms based on a scaled drive-by-wire chassis platform, as shown in Figure 8. The chassis was equipped with high-performance DC reduction motor as well as Ackerman steering motor in front axle, which utilized a controller area network (CAN) bus to communicate between various vehicle components. And the proposed algorithm was tested in three typical parking scenarios: parallel parking, perpendicular parking, and 60° angled parking.

For the velocity planning problem, the MATLAB built-in solver Quadprog 0.1.13, which deals with quadratic objective functions subject to linear constraints, was utilized for solving. The interior-point method was selected as the solving method. This method transforms a constrained optimization problem into an unconstrained one by introducing a barrier function and then continuously updates the utility function during the optimization iteration process to ensure algorithm convergence. For solving the ultimate optimal control problem, the MATLAB interface of ACADO Toolkit was employed. ACADO is a software environment and suite of algorithms for automatic control and dynamic optimization, written in C++, that provides a framework for direct optimal control using various algorithms. It also offers a MATLAB interface, importing ACADO’s capabilities for direct optimal control, model predictive control (MPC), and parameter estimation integrators and algorithms into MATLAB. The nonlinear optimization solver is generated by the ACADO code generation tool and implemented in a ROS (robot operating system) environment. The state variables are set as $x={\left[p_{x},p_{y},\varphi ,v,a,\delta ,\tau \right]}^{T}$, and the control variables as $u={\left[\omega ,j,\lambda ,\mu \right]}^{T}$. The related parameters for the optimization problem are set in Table 2.

### 4.1. Comparison of Trajectories Based on RS Paths and HC Paths

**Figure 9:**
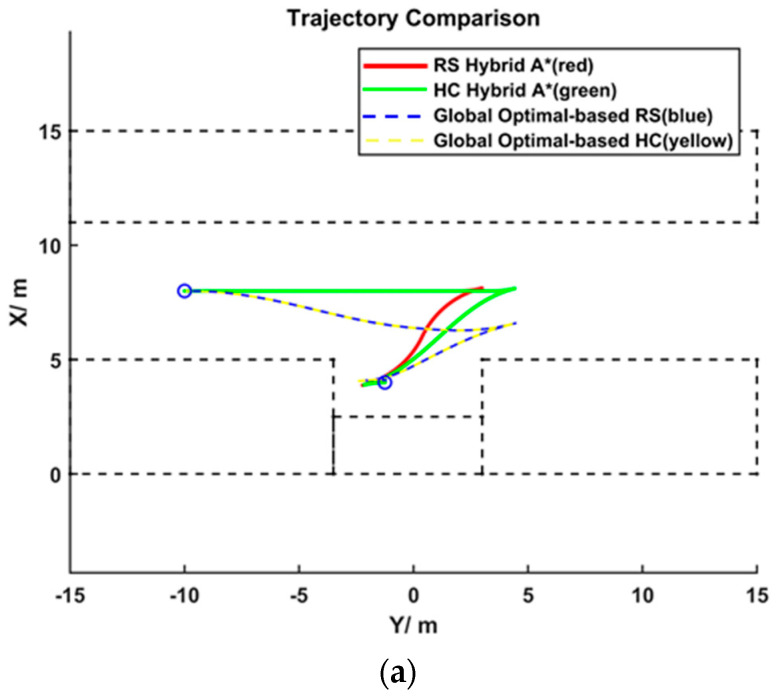

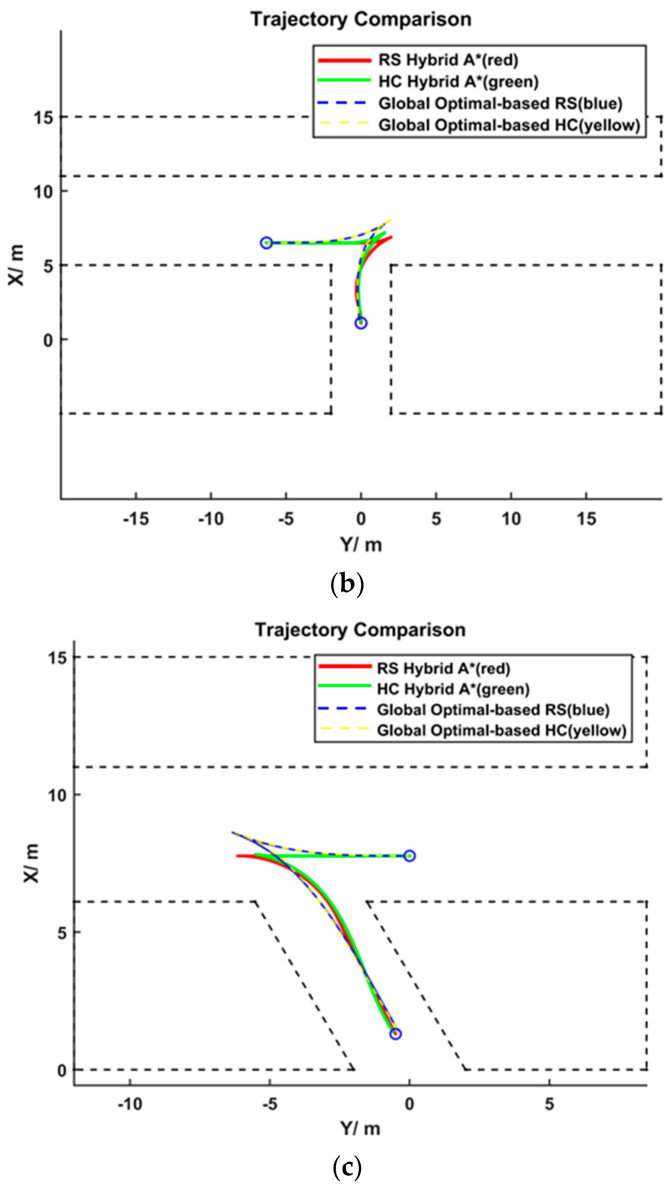
Comparison of trajectories based on RS paths and HC paths: the scenarios from left to right are parallel parking, perpendicular parking, and 60° angled parking. (**a**) parallel parking, (**b**) perpendicular parking, and (**c**) 60° angled parking.

Figure 9 presents the planning results based on RS paths and HC paths for different parking scenarios, and Table 3 provides a qualitative comparison of the planning results. It can be observed that in the parallel parking scenario, all planning results involved two gear shifts, while for perpendicular parking and angled parking, each planning result required only one shift. For the same scenario, the planned trajectories based on RS paths and HC paths involved the same number of gear shifts. Moreover, within the same scenario, the node positions where they ended the HA* search and proceeded to direct end-point connection were virtually identical, and the globally optimal outcomes were nearly indistinguishable when using these two different HA* results as initial values. The HC paths offer significant improvements over the RS paths in terms of curvature and rate of change of curvature. The HA* planning results based on HC paths were smoother, with fewer directional adjustments, making them closer to the globally optimal solution.

In this section, the path results of the improved HA* and the final optimization solution are analyzed and compared.

### 4.2. Optimal Trajectory Results

**Figure 10:**
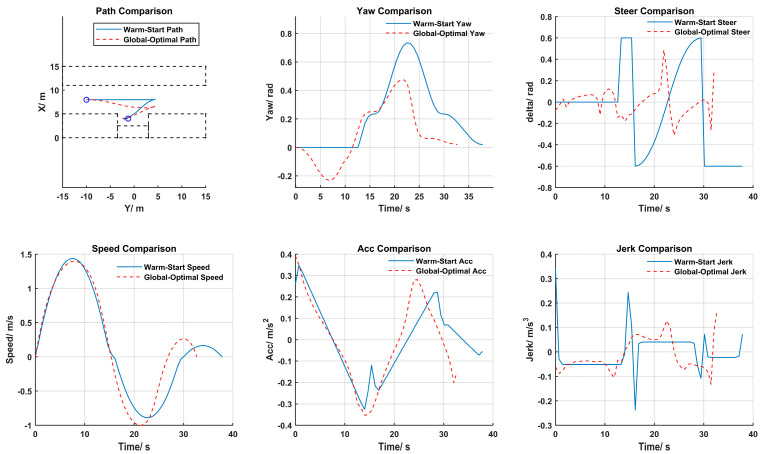
Parallel parking trajectory planning results.

**Figure 11:**
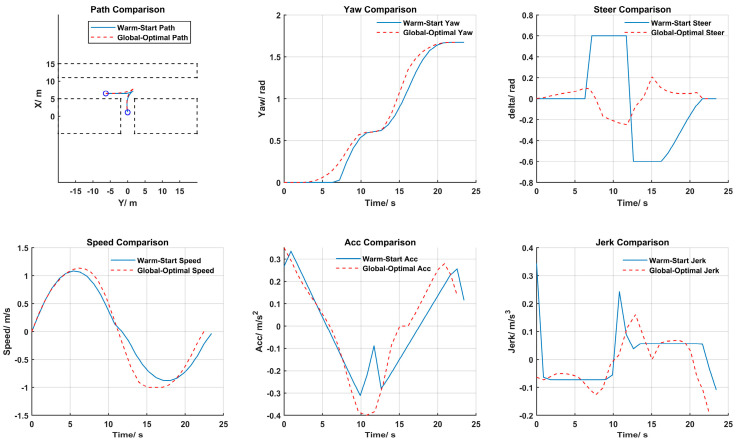
Perpendicular parking trajectory planning results.

**Figure 12:**
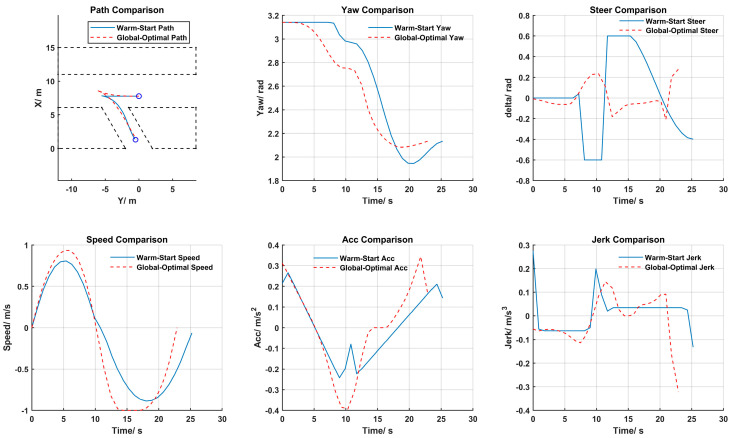
The 60° angled parking trajectory planning results.

The globally optimal trajectory solutions for the three parking scenarios are illustrated in Figure 10, Figure 11 and Figure 12. From top to bottom, from left to right, they sequentially display the path, heading, front wheel angle, speed, acceleration, and jerk for both the warm start and the globally optimal solution.

From the trajectory planning results of the parallel parking in Figure 10, it is known that the initial solution of the path’s warm start always searches along the straight line of the vehicle’s initial heading until it finds a collision-free HC path to connect to the endpoint. The whole process includes two gear shifts. The optimized path is closer to obstacles (other parking spaces) within a safe distance, therefore requiring a smaller parking space. The comparison of the heading angle and the front wheel angle indicates that the changes in the optimized path’s heading angle and front wheel angle were smoother, avoiding the on-the-spot turning problem found in the warm start initial solution for the front wheel angle, which can reduce tire wear. The velocity warm start initial solution itself was quite smooth, and except for the overall reduction in parking time due to minimizing the parking time, the overall trend of the velocity optimized solution did not differ significantly from the initial solution. At the same time, the acceleration change of the optimized trajectory was smoother, and the jerk throughout the parking process was reduced, resulting in improved stability and comfort.

According to the trajectory planning results for perpendicular parking in Figure 11, it is evident that both the warm start initial solution and the optimized solution of the path had only one gear shift. Compared to parallel parking, it is easier to find a feasible solution in perpendicular parking. Moreover, the optimal trajectory was closer to the obstacle above, which slightly increases the parking space but optimizes the steering changes on the premise of ensuring safety, resulting in a smoother trajectory. Like parallel parking, the initial trajectory of perpendicular parking also exhibited sudden changes in heading angle and front wheel angle. However, after global optimization, the steering adjustments and the accelerations/decelerations during the parking process were smoother, reducing tire wear and enhancing ride comfort. Additionally, the optimized trajectory reduced the overall parking time while ensuring safety, comfort, and stability of parking, thereby improving driving efficiency and offering higher economic benefits.

According to the results of the 60° angled parking trajectory planning shown in Figure 12, the process of angled parking is like that of perpendicular parking, with only one gear shift occurring. Due to the relatively small changes in the path’s angle during angled parking, the warm start initial path and the globally optimal path are close to each other. Likewise, the optimized trajectory for angled parking effectively avoids the issues of abrupt changes in angles and accelerations found in the initial solution, thereby reducing the total time required for parking.

To validate the computational efficiency, the experiment was run with an Intel(R) Core(TM) i7-8565U CPU @ 1.80 GHz x 4 processor (Intel, Santa Clara, CA, USA) and 16 GB RAM. The optimization times with a warm start by Hybrid A* for parallel parking, perpendicular parking, and 60° angled parking trajectory planning were 5.302 s, 4.765 s, and 5.768 s, respectively. In contrast, the optimization times without a warm start by Hybrid A* were 7.208 s, 7.136 s, and 8.623 s, respectively. These results clearly demonstrate that using a warm start for the optimization variables significantly accelerates the problem-solving process.

## 5. Conclusions

This paper proposes a feasible solution for the trajectory planning of autonomous parking. Based on the improved Hybrid A* algorithm, using the vehicle kinematic model and the distance between convex sets applied for setting obstacle avoidance constraints, a globally optimal planned trajectory can be finally obtained. A real car test was conducted to validate the feasibility and generalizability of the proposed globally optimized numerical optimization method with improved warm start in parallel, perpendicular, and angled parking scenarios. Compared to classical algorithms, the proposed method achieved smoother parking paths and more realistic control, providing a more effective solution for parking trajectory planning and tracking. However, merely tracking the obtained trajectory does not satisfy the parking requirements in highly variable scenarios encountered in real-world scenarios. Future work will focus on real-world vehicle testing to validate the proposed parking algorithm including environment perceptions.

## Figures and Tables

**Table 1:** HC families.

Combinations Based on RS	Other Combinations
C|C|C	CCC
C|CC	C|SC
CC|C	CS|C
CSC	C|SC
CC|CC	_
C|CC|C	_
C|CSC	_
CSC|C	_
C|CSC|C	_

**Table 2:** Parameters related to the drive-by-wire platform.

Symbol	Parameter	Value
$\delta_{max}$	Maximum front wheel angle (rad)	0.6
$del\_\delta_{max}$	Maximum front wheel angle change rate (rad/s)	0.6
$v_{max\_forward}$	Maximum forward speed (m/s)	2
$v_{max\_back}$	Maximum reverse speed (m/s)	1
$a_{max}$	Maximum acceleration (${m/s}^{2}$)	0.4
$jerk_{max}$	Maximum jerk (${m/s}^{3}$)	0.4
Δt	Sampling time of waypoints (s)	0.6
*timeScale* ^1^	Variable step size optimization coefficient range	[0, 1.5]

^1^ Note: The variable step size optimization coefficient is equal to the ratio of the optimized sampling time interval to the initial sampling time, that is, $\tau_{k}=timeScale_{k}*\Delta t$.

**Table 3:** Quantitative comparison of trajectory-related characteristics in three scenarios.

	Path Length/m			Average Curvature			Maximum Curvature Change			Number of Gear Shifts		
HA* Path based on RS	20.94	15.08	14.99	0.11	0.14	0.09	0.83	0.98	0.42	2	1	1
HA* Path based on HC	23.26	14.22	13.98	0.06	0.12	0.08	0.56	0.26	0.28	2	1	1
Optimal Path	23.25	16.11	15.81	0.06	0.08	0.06	0.25	0.11	0.20	2	1	1

## Data Availability

The data presented in this study are available on request from the corresponding author.
